# Vascular Disease and Risk Stratification for Ischemic Stroke and All-Cause Death in Heart Failure Patients without Diagnosed Atrial Fibrillation: A Nationwide Cohort Study

**DOI:** 10.1371/journal.pone.0152269

**Published:** 2016-03-25

**Authors:** Line Melgaard, Anders Gorst-Rasmussen, Lars Hvilsted Rasmussen, Gregory Y. H. Lip, Torben Bjerregaard Larsen

**Affiliations:** 1 Aalborg Thrombosis Research Unit, Department of Clinical Medicine, Faculty of Health, Aalborg University, Aalborg, Denmark; 2 Unit of Clinical Biostatistics, Aalborg University Hospital, Aalborg, Denmark; 3 University of Birmingham Institute of Cardiovascular Sciences, City Hospital, Birmingham, United Kingdom; 4 Department of Cardiology, Aalborg University Hospital, Aalborg, Denmark; Universitätsklinikum des Saarlandes, GERMANY

## Abstract

**Background:**

Stroke and mortality risk among heart failure patients previously diagnosed with different manifestations of vascular disease is poorly described. We conducted an observational study to evaluate the stroke and mortality risk among heart failure patients without diagnosed atrial fibrillation and with peripheral artery disease (PAD) or prior myocardial infarction (MI).

**Methods:**

Population-based cohort study of patients diagnosed with incident heart failure during 2000–2012 and without atrial fibrillation, identified by record linkage between nationwide registries in Denmark. Hazard rate ratios of ischemic stroke and all-cause death after 1 year of follow-up were used to compare patients with either: a PAD diagnosis; a prior MI diagnosis; or no vascular disease.

**Results:**

39,357 heart failure patients were included. When compared to heart failure patients with no vascular disease, PAD was associated with a higher 1-year rate of ischemic stroke (adjusted hazard rate ratio [HR]: 1.34, 95% confidence interval [CI]: 1.08–1.65) and all-cause death (adjusted HR: 1.47, 95% CI: 1.35–1.59), whereas prior MI was not (adjusted HR: 1.00, 95% CI: 0.86–1.15 and 0.94, 95% CI: 0.89–1.00, for ischemic stroke and all-cause death, respectively). When comparing patients with PAD to patients with prior MI, PAD was associated with a higher rate of both outcomes.

**Conclusions:**

Among incident heart failure patients without diagnosed atrial fibrillation, a previous diagnosis of PAD was associated with a significantly higher rate of the ischemic stroke and all-cause death compared to patients with no vascular disease or prior MI. Prevention strategies may be particularly relevant among HF patients with PAD.

## Introduction

Heart failure (HF) is associated with an increased risk of ischemic stroke and mortality[1], for which vascular disease is also an established risk factor[2,3]. In the general population, vascular disease is associated with an increased risk of cardiovascular events[4,5]. However, currently there is a lack of research on risk of ischemic stroke and mortality among incident HF patients in sinus rhythm previously diagnosed with different manifestations of vascular disease. Estimating the risk of ischemic stroke and all-cause death among HF patients in sinus rhythm with vascular disease is an important step towards finding subgroups of HF patients who might benefit from thromboprophylaxis, as suggested in a recent study[6] which found a high risk of ischemic stroke and thromboembolism among HF patients without atrial fibrillation (AF).

Vascular disease is a broad term, including two common and severe diseases, that is, peripheral artery disease (PAD) and myocardial infarction (MI). HF is known to be complicated by comorbidities such as PAD and prior MI[7,8], and assessment of these two comorbidities in relation to ischemic stroke, mortality, and prevention is important in a HF setting. However, PAD and prior MI may not confer the same risk of ischemic stroke[9]. Accordingly, evaluation of the association between vascular disease and ischemic stroke risk in the HF population requires investigating PAD and prior MI separately, as previously done in other settings[9,10]. The task of identifying subgroups of patients with HF who are at a high risk of stroke is clinically highly relevant because many such strokes may be preventable, for example, by pharmacological thromboprophylaxis.

The objective of the present observational cohort study was to assess the prognostic value of a prior diagnosis of PAD or MI in relation to the risk of ischemic stroke and all-cause death in HF patients, using Danish nationwide administrative registry data. We hypothesized that in a population of incident HF patients without AF (and not taking a vitamin K antagonist to avoid issues with effect modification by anticoagulation therapy), a prior diagnosis of either PAD or MI would be associated with a higher risk of ischemic stroke and all-cause death, when compared to no vascular disease, also when taking into account concomitant cardiovascular risk factors of ischemic stroke. Second, we hypothesized that PAD and prior MI would not contribute equally to this risk, since a difference in risk of ischemic stroke and all-cause death has been observed in other cardiovascular settings[9].

## Methods

### Registry Data Sources

We used three different nationwide registries in this study: i) The Danish National Patient Registry[11] which has registered all hospital admissions along with diagnoses since 1977 and codes all diagnoses according to the 10^th^ revision of the International Classification of Diseases (ICD-10) since 1994; ii) The National Prescription Registry[12] which contains data on all prescriptions dispensed from Danish pharmacies since 1994, coded according to the Anatomical Therapeutic Chemical (ATC) Classification System; iii) The Danish Civil Registration System which holds information on date of birth, migration, vital status, date of death, and sex of all persons living in Denmark[13]. Data were linked via unique personal identification number used in all Danish national registries. All three registries were up to December 31^st^ 2012. These registries have previously been validated[11,12,14], and the diagnosis of HF, ischemic stroke, PAD and MI was found to be valid[14–16].

### Study Population

The study population was identified as in- or outpatients aged>50 years, diagnosed with a primary discharge diagnosis of incident (first-time diagnosis) HF in the period January 1^st^ 2000—December 31^st^ 2012 (ICD-10: I50, I42.0, I11.0, I13.0, I13.2). “Vascular disease” was defined as a diagnosis of either PAD (ICD-10: I70.2-I70.9, I73.9) or MI (ICD-10: I21.0-I21.9, I23.0-I23.9) between 1994 and time of HF diagnosis. Patients with prior diagnoses of both PAD and MI were not included in the analyses: this is a small and seriously ill patient group, against which direct comparisons would have both limited statistical power and clinical use. To restrict to patients without AF, we excluded those who had a prior diagnosis of AF or atrial flutter (I48) between 1994 and time of HF diagnosis. We moreover excluded patients treated with a vitamin K antagonist (ATC: B01AA03, B01AA04) within six months prior to the HF diagnosis to avoid considering effect modification by anticoagulation therapy. Lastly, patients with a diagnosis of cancer (ICD-10: C00-C97) within 5 years before HF diagnosis were excluded, since cancer patients represents a subgroup with high stroke risk[17] and specialized thromboprophylactic treatment regimens.

Additional comorbidities were assessed at time of HF diagnosis identified using the Danish National Patient Registry and the Danish National Prescription Registry which have registered diagnoses (using ICD-10) and prescriptions since 1994. Ascertainment of baseline medication status was based on medication purchase in a 45-day window before or after the date of HF diagnosis. ICD-codes and ATC-codes used to define comorbidities and medical therapy are provided in the supporting materials [please see table in S1 Table in the supporting materials].

### Outcomes

The primary endpoint was defined as an ischemic stroke diagnosis (ICD-10: I63, I64). Because of the high mortality in the HF population and the fact that in registries some deaths may be due to undiagnosed stroke, especially since postmortems and cerebral scanning are not mandated, all-cause death was also included as a primary endpoint.

### Statistical Methods

Baseline characteristics were described separately for patients with no vascular disease, PAD, and prior MI; using means and standard deviation for continuous variables, and proportions for categorical variables.

Time-to-event analysis was used to describe the association with the three-level vascular disease exposure (no vascular disease; prior PAD; prior MI) and risk of ischemic stroke or all-cause death. Time at risk was measured from baseline date (date of HF diagnosis) and until an event of ischemic stroke, all-cause death, emigration, or end of study (December 31^st^ 2012), whichever came first. Additionally, patients were censored if they initiated anticoagulant therapy during the follow-up period.

We have previously advocated risks (probabilities) rather than rates for assessing associations in a HF population, since risks lead to statements which are more clinically and prognostically relevant when faced with a high competing mortality risk[18]. However, strong differential competing mortality across exposure levels can lead to counterintuitive findings on a risk scale. This is a real concern for vascular disease as an exposure. Accordingly, we reported associations in terms of (Cox model) hazard rate ratios after 1 year of follow-up. Following the suggestions of Andersen et al.[19] to consider both risk and rate assessments, we repeated this analysis on a risk (ratio) scale; see text in S1 Text in the supporting materials for methodological details.

Specifically, for the primary analysis, Cox regression was used to calculate 1-year hazard rate ratios of the endpoints according to the presence of PAD or prior MI (5-year hazard ratios are reported in the supporting materials). Concomitant baseline comorbidities such as hypertension, diabetes, renal disease, and prior ischemic stroke are well-known risk factors of ischemic stroke in other heart diseases[20,21]. Thus, in order to assess the independent prognostic value of PAD and prior MI, we also fitted Cox models after adjusting for these risk factors; alongside with chronic obstructive pulmonary disease (COPD), age and sex. Antiplatelet therapy may modify the association between vascular disease and ischemic stroke risk; therefore, we calculated 1-year hazard rate ratios of the endpoints according to the presence of PAD or prior MI, and stratified by antiplatelet therapy at baseline (5-year hazard ratios are reported in the supporting materials).

We performed sensitivity analysis in which we censored patients receiving a diagnosis of AF during follow-up. We also performed a sensitivity analysis in which patients with a history of stroke were excluded (since a prior stroke diagnosis is a strong risk factor for a subsequent stroke)[22]. Additionally, crude cumulative incidence curves for both end points according to vascular disease were constructed based on the Aalen-Johansen estimator for competing risks data.

The analyses were performed using Stata version 13 (Stata Corporation, College Station, TX, USA). A two-sided p-value of <0.05 was considered statistically significant. The study was conducted and reported in accordance with the Strengthening the Reporting of Observational Studies in Epidemiology (STROBE) recommendations.

### Ethical Considerations

No ethical approval is required for anonymous register studies in Denmark. The study was approved by the Danish Data Protection Agency (J. No. File No. 2012-41-0633).

## Results

**Fig 1:**
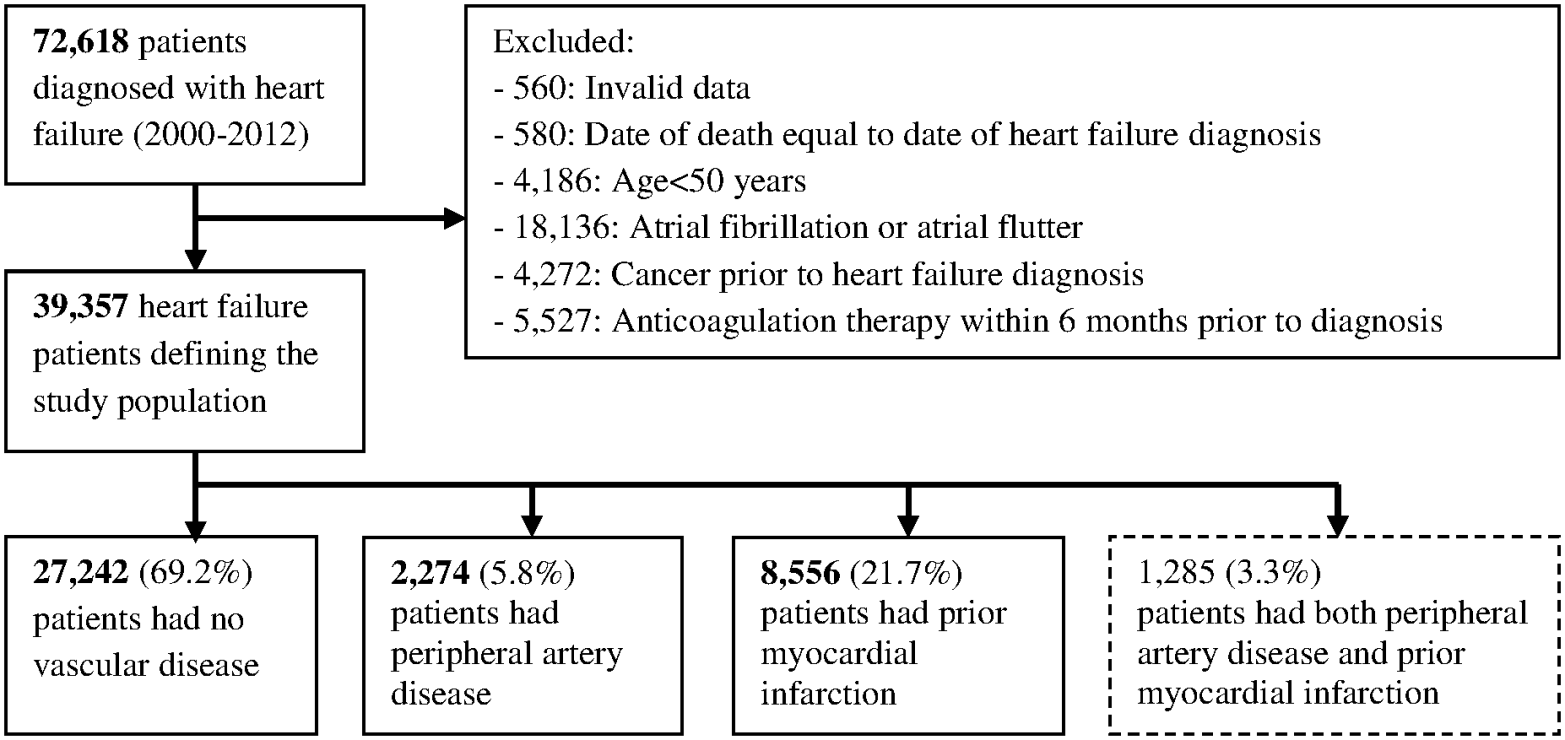
Flowchart of patients included in the final study population.

The study population comprised 39,357 heart failure patients aged>50 years, among which 69.2% had no vascular disease, 5.8% had PAD and 21.7% had prior MI (3.3% had both) [please see Fig 1]. The median follow-up time for ischemic stroke or all-cause death was 2.5 years (interquartile range 0.6–5.3).

Baseline patient characteristics are summarized in Table 1. The mean age among patients with no vascular disease was 74.3 years (standard deviation (SD): 11.7) compared to 74.9 years (SD: 9.9) and 73.1 years (SD: 10.9) years for patients with PAD and prior MI, respectively. Patients with PAD had more often experienced a previous stroke or transient ischemic attack compared to both patients with prior MI or no vascular disease, and the prevalence of diabetes, hypertension, renal disease, and COPD was higher in patients with PAD. More patients with prior MI were taking medical therapy with ACE-inhibitors, beta-blockers and statins. Furthermore, almost 75% of the patients with prior MI were on antiplatelet therapy, compared to around 50% of the patients with PAD.

**Table 1:** Baseline characteristics of study population, stratified according to vascular disease.

Clinical characteristics	No vascular disease	Peripheral artery disease	Prior myocardial infarction
N, % (n)	69.2 (27,242)	5.8 (2,274)	21.7 (8,556)
Sex (females), % (n)	48.4 (13,191)	45.3 (1,030)	33.0 (2,824)
Average age at baseline, years (SD)	74.3 (11.7)	74.9 (9.9)	73.1 (10.9)
*Baseline comorbidity*, *% (n)*			
Previous stroke/transient ischemic attack	11.2 (3,060)	22.3 (507)	14.1 (1,203)
Diabetes	12.0 (3,264)	23.1 (525)	16.1 (1,374)
Hypertension	28.5 (7,752)	44.5 (1,012)	35.0 (2,991)
Renal Disease	4.5 (1,215)	10.0 (228)	5.8 (495)
Liver Disease	0.5 (136)	0.4 (9)	0.3 (29)
Hyperthyroidism	2.7 (723)	3.7 (83)	2.1 (182)
COPD	13.3 (3,632)	19.7 (447)	11.2 (959)
*Baseline medication*, *% (n)*			
ACE-inhibitors	47.9 (13,040)	46.5 (1,058)	62.5 (5,345)
Angiotensin receptor blocker	10.0 (2,721)	12.8 (292)	11.0 (941)
Beta-blockers	37.2 (10,138)	36.8 (837)	64.3 (5,497)
Aldosterone antagonists	22.9 (6,225)	22.7 (516)	23.3 (1,990)
Non-loop diuretics	40.0 (10,902)	40.0 (910)	35.5 (3,034)
Loop diuretics	66.2 (18,041)	70.8 (1,609)	60.6 (5,185)
Statins	21.0 (5,732)	34.0 (772)	57.1 (4,888)
NSAIDs	14.6 (3,973)	14.4 (327)	12.3 (1,048)
Aspirin	40.5 (11,032)	49.7 (1,129)	68.4 (5,851)
Thienopyridines	3.8 (1,043)	6.4 (145)	31.6 (2,703)

(Abbreviations: COPD = chronic obstructive pulmonary disease; NSAIDs = non-steroidal anti-inflammatory drugs; SD = standard deviation)

The number of ischemic strokes and deaths in each patient group after 1 year of follow-up are shown in Table 2. For patients with no vascular disease, PAD, or prior MI, the absolute risks of both endpoints are shown in Table 2. The 1-year absolute risks of ischemic stroke and all-cause death were highest in patients with PAD (4.7% and 30.8%, respectively). For the 5-year event numbers and absolute risks please see table in S2 Table in the supporting materials.

**Table 2:** Event numbers and absolute risks of ischemic stroke and all-cause death after 1-year follow-up, according to vascular disease.

ENDPOINT	Event number	Absolute risk*, %
**Ischemic stroke**		
No vascular disease	705	2.7
PAD	101	4.7
Prior MI	250	3.0
**All-cause death**		
No vascular disease	5486	21.5
PAD	661	30.8
Prior MI	1509	18.5

* Taking into account competing risks of death (Aalen-Johansen estimator).

(Abbreviations: MI: myocardial infarction; PAD: peripheral artery disease)

In the Cox regression analysis, when compared to HF patients with no vascular disease, PAD was associated with a higher 1-year rate of ischemic stroke (adjusted hazard rate ratio [HR]: 1.34, 95% confidence interval [CI]: 1.08 to 1.65) and all-cause death (adjusted HR: 1.47, 95% CI: 1.35 to 1.59), whereas prior MI was not (adjusted HR: 1.00, 95% CI: 0.86 to 1.15 and 0.94, 95% CI: 0.89 to 1.00, for ischemic stroke and all-cause death, respectively), as shown in Table 3. When comparing patients with PAD to patients with prior MI, PAD was associated with a higher rate of both endpoints (adjusted HR: 1.36, 95% CI: 1.07 to 1.72 and 1.53, 95% CI: 1.40 to 1.68, for ischemic stroke and all-cause death, respectively). Similar results were obtained after 5-years follow-up [please see table in S3 Table in the supporting materials]. Cumulative incidence curves for both end points demonstrate a steady increase during follow-up [please see figure in S1 Fig in the supporting materials].

**Table 3:** Hazard rate ratios of ischemic stroke and all-cause death after 1-year follow-up, according to vascular disease.

ENDPOINT	PRIMARY EFFECT ESTIMATES			
**Ischemic stroke**	**Crude HR**	**(95% CI)**	Adjusted HR*	**(95% CI)**
PAD vs. no vascular disease	1.82	(1.47 to 2.24)	1.34	(1.08 to 1.65)
Prior MI vs. no vascular disease	1.09	(0.94 to 1.26)	1.00	(0.86 to 1.15)
PAD vs. prior MI	1.67	(1.32 to 2.10)	1.36	(1.07 to 1.72)
**All-cause death**	**Crude HR**	**(95% CI)**	Adjusted HR*	**(95% CI)**
PAD vs. no vascular disease	1.51	(1.40 to 1.64)	1.47	(1.35 to 1.59)
Prior MI vs. no vascular disease	0.85	(0.80 to 0.90)	0.94	(0.89 to 1.00)
PAD vs. prior MI	1.78	(1.63 to 1.95)	1.53	(1.40 to 1.68)

(Abbreviations: HR: hazard ratio; MI: myocardial infarction; PAD: peripheral artery disease; 95% CI: 95% confidence interval)

*Adjusted for sex (binary), hypertension (binary), diabetes (binary), prior stroke/transient ischemic attack (binary), COPD (binary), renal disease (binary), and age (continuous)

The stratified HRs of ischemic stroke and all-cause death after 1-year follow-up, according to antiplatelet therapy at baseline, are shown in Table 4. Regardless of antiplatelet therapy status at baseline, PAD was associated with a higher 1-year rate of ischemic stroke and all-cause death when compared to HF patients with no vascular disease or prior MI. In patients on antiplatelet therapy, we found similar rates of ischemic stroke and all-cause death among patients with prior MI compared to those with no vascular disease. In patients not on antiplatelet therapy, prior MI was associated with an increased rate of all-cause death, when compared to no vascular disease. Similar results were obtained after 5-years follow-up [please see table in S4 Table in the supporting materials].

**Table 4:** Hazard rate ratios of ischemic stroke and all-cause death after 1-year follow-up, stratified by antiplatelet therapy at baseline.

ENDPOINT	STRATIFIED ADJUSTED ESTIMATES*			
	No antiplatelet therapy		Antiplatelet therapy	
**Ischemic stroke**	HR	(95% CI)	HR	(95% CI)
PAD vs. no vascular disease	1.49	(1.10 to 2.01)	1.23	(0.91 to 1.65)
Prior MI vs. no vascular disease	1.03	(0.79 to 1.34)	1.02	(0.85 to 1.22)
PAD vs. prior MI	1.45	(1.00 to 2.12)	1.23	(0.90 to 1.68)
	No antiplatelet therapy		Antiplatelet therapy	
**All-cause death**	HR	(95% CI)	HR	(95% CI)
PAD vs. no vascular disease	1.43	(1.29 to 1.60)	1.65	(1.45 to 1.86)
Prior MI vs. no vascular disease	1.27	(1.17 to 1.38)	0.99	(0.91 to 1.07)
PAD vs. prior MI	1.11	(0.98 to 1.26)	1.64	(1.44 to 1.87)

(Abbreviations: MI: myocardial infarction; PAD: peripheral artery disease; HR: hazard ratio; 95% CI: 95% confidence interval)

*Adjusted for sex (binary), hypertension (binary), diabetes (binary), prior stroke/transient ischemic attack (binary), COPD (binary), renal disease (binary), and age (continuous)

The 1- and 5-year relative risks of ischemic stroke and all-cause death, comparing patients with PAD or prior MI to patients with no vascular disease are shown in tables in S7 and S8 Tables in the supporting materials. We found similar crude associations as in the rate-based calculations, but the associations attenuated after adjustment for cardiovascular risk factors, especially for the endpoint of ischemic stroke.

### Sensitivity analyses

In the sensitivity analysis in which patients were censored when they were diagnosed with AF during follow-up, findings were similar to the main analysis [please see table in S5 Table in the supporting materials]. When excluding patients with a history of stroke, PAD was associated with an even higher rate of ischemic stroke in the adjusted analyses compared to patients with no vascular disease and prior MI [please see table in S6 Table in the supporting materials].

## Discussion

In this large nationwide cohort study of HF patients without AF, we found a significantly higher rate of ischemic stroke and all-cause death amongst HF patients with PAD compared to patients with no vascular disease or prior MI, even after extensive adjustment for concomitant cardiovascular risk factors. Similar results were obtained in the stratified analysis, regardless of antiplatelet therapy. However, for the risk (probability) based calculations, the picture was less consistent.

**To our knowledge, this is the first study to comprehensively evaluate the prognostic value of PAD and prior MI in relation to ischemic stroke and all-cause death in a HF population without diagnosed AF.** The current evidence on the risk of ischemic stroke in subgroups of HF patients without diagnosed AF is very limited[23–25]. In our population, PAD was associated with a roughly 30% higher rate of ischemic stroke compared to patients without vascular disease. We did not see a similar increased rate of ischemic stroke among patients with prior MI, as expected. Indeed, we found PAD to be associated with a higher rate of ischemic stroke compared to prior MI.

Our findings for the rate of stroke and mortality are consistent with the possibility of less secondary prevention strategies in PAD patients compared to MI patients, as seen in other settings[26,27]. In our study, a larger proportion of patients with prior MI were taking medical therapy with ACE-inhibitors, beta-blockers, statins, and antiplatelet therapy compared to patients with PAD. This was despite patients with PAD having more comorbidities, such as prior stroke/transient ischemic attack, diabetes, and hypertension. The lower rate of ischemic stroke and all-cause death among prior MI patients compared to PAD patients may reflect a more ill patients group of those with PAD[28]. Alternatively, these findings may reflect a more intensified treatment and prophylaxis among patients with coronary artery disease[27]. For example, antiplatelet therapy is known to reduce the risk of ischemic stroke[29,30]. Our findings in the subpopulation of patients on antiplatelet therapy were consistent with the hypothesis of more intensified treatment and prophylaxis among MI patients, since patients on antiplatelet therapy with prior MI or no vascular disease had essentially the same rate of ischemic stroke.

Patients with PAD are likely to have concomitant coronary artery disease or cerebrovascular disease, which from an etiological perspective could influence the association between vascular disease and stroke risk; however, since the focus of the study was on the *prognostic value* of PAD and prior MI in relation to ischemic stroke, and not to elucidate a potential causal role, confounding is per definition not an issue of concern. Hence, our observations of a higher stroke risk in patients with PAD versus no vascular disease cannot exclusively be attributed to an *effect* of PAD, but is likely to be explained also by other concomitant stroke risk factors, such as lifestyle factors (e.g., smoking), coexisting cerebrovascular disease, and etiology and subtype of HF. For example, coronary artery disease is an important cause of HF, and we therefore possibly compared patient populations with different underlying substrates for HF. If our aim had been to examine the *effect* of vascular disease on stroke risk, this would have required a different study design with use of a more broad definition of vascular disease, taking into account possible confounding stroke risk factors, but this was beyond the scope of this study which had focus on stroke risk stratification in the HF population.

### Clinical implications

Reduced secondary prevention in PAD patients compared to patients with coronary artery disease has been observed in several studies[26,27]; notwithstanding guideline recommendations that the same secondary prevention should be used in patients with PAD and prior MI[31,32]. Our results provide some indication that, in clinical practice, HF patients with PAD may represent a higher-risk subgroup in terms of ischemic stroke and all-cause death risk compared to prior MI patients. It is possible that more focus on secondary prevention could improve prognosis for this patient group. Currently, an ongoing trial (COMMANDER HF) is exploring the efficacy and safety of rivaroxaban (one of the non-vitamin K antagonist oral anticoagulants) compared with placebo (standard care) after an exacerbation of HF in non-AF patients with HF with reduced ejection fraction and documented coronary artery disease. However, whether full anticoagulation therapy would be beneficial in HF patients with PAD is an open question in need of further investigation before recommendations about changes in management of these patients can be given, since recent prospective randomized controlled trials of antithrombotic therapy in HF have not investigated such subgroup issues[33–36].

We provided both risk/probability (risk ratio) and rate (hazard ratio) assessments of associations[19]. While risk and rate assessments are traditionally thought of as being equivalent, they can be fundamentally different in the face of competing mortality risk[37]. In the present study, associations were attenuated when viewed on a risk scale. This is important information from a clinical perspective, since it may indicate a smaller absolute potential (e.g. in terms of number of strokes prevented) of prevention strategies among PAD patients than otherwise suggested by the hazard ratios.

### Strengths and Limitations

The major strengths of this study are the validated outcomes and large sample size uniquely possible with this type of cohort study. Selection into the study was not an issue, since we investigated a nationwide population cohort of incident HF patients without AF, with limited loss to follow-up.

The study also has several important limitations. We were unable to distinguish between HF with preserved and reduced ejection fraction or estimate the functional classification, since we did not have access to echocardiograms. However, no difference in embolic risk (risk of stroke, transient ischemic attack, systemic embolism) was found in a recent study of non-anticoagulated patients with HF with reduced or preserved ejection fraction[38]. Similarly, in a post-hoc analysis of a study of AF patients with HF with reduced or preserved ejection fraction, no difference in ischemic stroke risk was found between the groups[39]. The diagnosis of HF has previously been validated and had a positive predictive value of 81–100%[16,40]. Based on the validation study, we did not capture all patients with HF and also cannot be certain that all patients identified as having HF had definite HF, which could lead to imprecision in the risk estimates. However, we included only patients with a primary discharge diagnosis of HF to optimize the probability of including only correctly identified patients with HF. We excluded HF patients younger than 50 years of age, and accordingly, our findings may not apply to younger HF patients.

The exposure variable of vascular disease may also be subject to misclassification and incomplete ascertainment. Moreover, we did not have information about the ankle-brachial index, and were hence unable to use this measurement in the definition of PAD.

We included unspecified stroke (ICD-10: I64) in the definition of ischemic stroke, as most strokes are of ischemic origin. However, we cannot rule out that some of these strokes might have been hemorrhagic strokes and thus, misclassified as ischemic strokes. Also, we cannot rule out that some patients might have had undiagnosed AF, since heart disease is associated with an increased risk of developing AF, however, censoring for presence of AF during follow-up did not change the main conclusions.

Last, the study was carried as a nationwide study in the Danish population, which both ethnically and socioeconomically is fairly homogeneous. Future studies are needed to evaluate if our findings hold in more diverse populations.

## Conclusion

Among incident HF patients without AF prior PAD was associated with a significantly higher rate of ischemic stroke and all-cause death compared to both patients with no vascular disease and with prior MI, even after adjustment for concomitant cardiovascular risk factors.

## Supporting Information

S1 Fig Cumulative incidence of ischemic stroke and all-cause death: A) Ischemic stroke; B) All-cause death. (DOCX)

S1 Table ICD-10 codes and ATC-codes used in the cohort study. (DOCX)

S2 Table Event numbers and absolute risks of ischemic stroke and all-cause death after 5-years follow-up, according to vascular disease. (DOCX)

S3 Table Hazard rate ratios of ischemic stroke and all-cause death after 5-years follow-up, according to vascular disease. (DOCX)

S4 Table Hazard rate ratios of ischemic stroke and all-cause death after 5-year follow-up, stratified by antiplatelet therapy at baseline. (DOCX)

S5 Table Sensitivity analysis censoring patients diagnosed with AF during follow-up: Hazard rate ratios of ischemic stroke and all-cause death after 1-year follow-up, according to vascular disease. (DOCX)

S6 Table Sensitivity analysis excluding patients with a history of stroke: Hazard rate ratios of incident stroke after 1-year follow-up, according to vascular disease. (DOCX)

S7 Table Relative risks of ischemic stroke and all-cause death after 1-year follow-up, according to vascular disease. (DOCX)

S8 Table Relative risks of ischemic stroke and all-cause death after 5-years follow-up, according to vascular disease. (DOCX)

S1 Text Methodological details- Analysis on a risk-scale. (DOCX)

## Abbreviations

AF: atrial fibrillation
COPD: chronic obstructive pulmonary disease
HF: heart failure
MI: myocardial infarction
PAD: peripheral artery disease
