# Supplementary material for: Vascular Disease and Risk Stratification for Ischemic Stroke and All-Cause Death in Heart Failure Patients without Diagnosed Atrial Fibrillation: A Nationwide Cohort Study
Source: PLoS One. 2016 Mar 25;11(3):e0152269. doi: 10.1371/journal.pone.0152269 (PMC4807813; doi:10.1371/journal.pone.0152269)
Supplement: S1 Fig — (DOCX) [file pone.0152269.s001.docx]

**S1 Fig.** Cumulative incidence of ischemic stroke and all-cause death:

A) Ischemic stroke; B) All-cause death.

A

B
