# Supplementary material for: Vascular Disease and Risk Stratification for Ischemic Stroke and All-Cause Death in Heart Failure Patients without Diagnosed Atrial Fibrillation: A Nationwide Cohort Study
Source: PLoS One. 2016 Mar 25;11(3):e0152269. doi: 10.1371/journal.pone.0152269 (PMC4807813; doi:10.1371/journal.pone.0152269)
Supplement: S1 Table — (DOCX) [file pone.0152269.s002.docx]

**S1 Table.** ICD-10 codes and ATC-codes used in the cohort study.

| **ICD 10-Codes and ATC-Codes used in the Study** | |
| --- | --- |
| **Main diagnosis** | **ICD 10-Codes** |
| Congestive heart failure | I50.0-I50.9, I11.0, I13.0, I13.2 |
| Acute myocardial infarction | I21.0-I21.9, I23.0-I23.8 |
| Peripheral arterial disease* | I70.2-I70.9, I73.9 |
| **Endpoint** |  |
| Stroke (ischemic) | I63.0-I63.9, I64 |
| **Comorbidities** | **ICD 10-Codes** |
| Prior stroke (ischemic or hemorrhagic) / transient ischemic attack | I60.0-I60.9, I61.0-I61.9, I62.0-I62.9, I63.0-I63.9, I64.9, G45† |
| Vascular disease | I21.0-I21.9, I23.0-I23.9, I70.0, I70.2-I70.9, I73.9 |
| Diabetes mellitus | E10.0-E10.9, E11.0-E11.9 |
| Hypertension | I10.0-I10.9, I11.0-I11.9, I12.0-I12.9, I13.0-I13.9, I15.0–I15.9 |
| Renal disease | I12.0-I12.9, I13.0-I13.9, N00-N07, N11.0-N11.9, N14.0-N14.4, N17.0-N17.9, N18.0-N18.9, N19, Q61.0-Q61.9 |
| Liver disease | B15.0-B15.9, B16.0-B16.9, B17.0-B17.9, B18.0-B18.9, B19.0-B19.9, K70.4, K72.0-K72.9, K76.6 |
| Hyperthyroidisme | E05.0-E05.9, E06.0-E06.9 |
| Chronic obstructive pulmonary disease (COPD) | J44.0-J44.9 |
| Atrial fibrillation and flutter (exclusion criteria) | I48 |
| Cancer any type (exclusion criteria) | C00-C97 |
| **Concomitant medication** | **ATC-Codes** |
| Warfarin (exclusion criteria) | B01AA03 |
| Phenprocoumon (exclusion criteria) | B01AA04 |
| ACE-inhibitors | C09AA |
| Angiotension receptor blockers | C09CA |
| Beta-blockers | C07 |
| Non-loop diuretics | C02DA, C02L, C03A, C03B, C03D, C03E, C03X, C07C, C07D, C08G,C09BA, C09DA, C09XA52 |
| Aldosterone antagonists | C03DA |
| Loop diuretics | C03C |
| Statins | C10 |
| Non steroidal anti-inflammatory drugs (NSAIDs) | M01A |
| Aspirin | B01AC06 |
| Thienopyridines | B01AC04, B01AC22, B01AC24 |
|  | |
| **Peripheral arterial disease, refers to the obstruction of large arteries not within the coronary, aortic arch vasculature, or brain*  *†Not inclusive G45.3 (Amaurosis fugax)* | |
