# Supplementary material for: Vascular Disease and Risk Stratification for Ischemic Stroke and All-Cause Death in Heart Failure Patients without Diagnosed Atrial Fibrillation: A Nationwide Cohort Study
Source: PLoS One. 2016 Mar 25;11(3):e0152269. doi: 10.1371/journal.pone.0152269 (PMC4807813; doi:10.1371/journal.pone.0152269)
Supplement: S2 Table — (DOCX) [file pone.0152269.s003.docx]

| **ENDPOINT** | | | **Event number** | | | **Absolute risk*, %** | |
| --- | --- | --- | --- | --- | --- | --- | --- |
|  | | |  | |  |  |  |
| **Ischemic stroke** | |  | |  |  |  |  |
|  | No vascular disease |  | |  | 1559 |  | 6.8 |
|  | PAD |  | |  | 185 |  | 9.2 |
|  | Prior MI |  | |  | 569 |  | 7.9 |
| **All-cause death** | |  | |  |  |  |  |
|  | No vascular disease |  | |  | 10805 |  | 48.5 |
|  | PAD |  | |  | 1239 |  | 66.1 |
|  | Prior MI |  | |  | 2977 |  | 42.9 |
|  |  |  | |  |  |  |  |
| (Abbreviations: MI: myocardial infarction; PAD: peripheral artery disease)  * Taking into account competing risks of death (Aalen-Johansen estimator). | | | | | | | |

**S2 Table.** Event numbers and absolute risks of ischemic stroke and all-cause death after 5-years follow-up, according to vascular disease.
