# Supplementary material for: Vascular Disease and Risk Stratification for Ischemic Stroke and All-Cause Death in Heart Failure Patients without Diagnosed Atrial Fibrillation: A Nationwide Cohort Study
Source: PLoS One. 2016 Mar 25;11(3):e0152269. doi: 10.1371/journal.pone.0152269 (PMC4807813; doi:10.1371/journal.pone.0152269)
Supplement: S3 Table — (DOCX) [file pone.0152269.s004.docx]

**S3 Table.** Hazard rate ratios of ischemic stroke and all-cause death after 5-years follow-up, according to vascular disease.

| **ENDPOINT** | | **PRIMARY EFFECT ESTIMATES** | | | | | |
| --- | --- | --- | --- | --- | --- | --- | --- |
| **Ischemic stroke** | | **Crude HR**  **(95% CI)** | | | | **Adjusted HR***  **(95% CI)** | |
|  | |  |  | | |  |  |
|  | PAD vs. no vascular disease | 1.68 | (1.44 to 1.96) | | | 1.32 | (1.13 to 1.54) |
|  | Prior MI vs. no vascular disease | 1.11 | (1.01 to 1.22) | | | 1.04 | (0.94 to 1.15) |
|  | PAD vs. prior MI | 1.56 | (1.28 to 1.79) | | | 1.23 | (1.04 to 1.46) |
|  |  |  |  | | |  |  |
| **All-cause death** | | **Crude HR**  **(95% CI)** | | | | **Adjusted HR***  **(95% CI)** | |
|  | |  |  | | |  |  |
|  | PAD vs. no vascular disease | 1.60 | (1.51 to 1.70) | | | 1.52 | (1.43 to 1.61) |
|  | Prior MI vs. no vascular disease | 0.84 | (0.81 to 0.87) | | | 0.91 | (0.88 to 0.95) |
|  | PAD vs. prior MI | 1.91 | (1.79 to 2.04) | | | 1.63 | (1.53 to 1.75) |
|  |  |  |  |  |  | | |
| (Abbreviations: HF: heart failure; HR: hazard rate ratio; MI: myocardial infarction; PAD: peripheral artery disease; 95% CI: 95% confidence interval)  *Adjusted for sex (binary), hypertension (binary), diabetes (binary), prior stroke/transient ischemic attack (binary), COPD (binary), renal disease (binary), and age (continuous) | | | | | | | |
