# Supplementary material for: Vascular Disease and Risk Stratification for Ischemic Stroke and All-Cause Death in Heart Failure Patients without Diagnosed Atrial Fibrillation: A Nationwide Cohort Study
Source: PLoS One. 2016 Mar 25;11(3):e0152269. doi: 10.1371/journal.pone.0152269 (PMC4807813; doi:10.1371/journal.pone.0152269)
Supplement: S4 Table — (DOCX) [file pone.0152269.s005.docx]

**S4 Table**: Hazard rate ratios of ischemic stroke and all-cause death after 5-year follow-up, stratified by antiplatelet therapy at baseline.

| **ENDPOINT** | | **STRATIFIED ADJUSTED ESTIMATES*** | | | | | |
| --- | --- | --- | --- | --- | --- | --- | --- |
| **Ischemic stroke** | | **No antiplatelet therapy**  **HR (95% CI)** | | **Antiplatelet therapy**  **HR (95% CI)** | | | |
|  | |  |  |  |  | |  |
|  | PAD vs. no vascular disease | 1.51 | (1.21 to 1.87) | 1.18 | (0.95 to 1.47) | |  |
|  | Prior MI vs. no vascular disease | 1.16 | (0.98 to 1.38) | 1.05 | (0.93 to 1.18) | |  |
|  | PAD vs. prior MI | 1.25 | (0.96 to 1.62) | 1.11 | (0.88 to 1.39) | |  |
|  |  |  |  |  |  | |  |
| **All-cause death** | | **No antiplatelet therapy**  **HR (95% CI)** | | **Antiplatelet therapy**  **HR (95% CI)** | | | |
|  | |  |  |  |  | |  |
|  | PAD vs. no vascular disease | 1.48 | (1.36 to 1.60) | 1.65 | (1.51 to 1.79) | |  |
|  | Prior MI vs. no vascular disease | 1.14 | (1.07 to 1.22) | 0.95 | (0.90 to 1.00) | |  |
|  | PAD vs. prior MI | 1.27 | (1.15 to 1.41) | 1.72 | (1.57 to 1.88) | |  |
|  |  |  |  |  |  | |  |
| (Abbreviations: HF: heart failure; MI: myocardial infarction; PAD: peripheral artery disease; HR: hazard ratio; 95% CI: 95% confidence interval)  *Adjusted for sex (binary), hypertension (binary), diabetes (binary), prior stroke/transient ischemic attack (binary), COPD (binary), renal disease (binary), and age (continuous) | | | | | |  |  |
