# Supplementary material for: Vascular Disease and Risk Stratification for Ischemic Stroke and All-Cause Death in Heart Failure Patients without Diagnosed Atrial Fibrillation: A Nationwide Cohort Study
Source: PLoS One. 2016 Mar 25;11(3):e0152269. doi: 10.1371/journal.pone.0152269 (PMC4807813; doi:10.1371/journal.pone.0152269)
Supplement: S5 Table — (DOCX) [file pone.0152269.s006.docx]

**S5 Table.** Sensitivity analysis censoring patients diagnosed with AF during follow-up: Hazard rate ratios of ischemic stroke and all-cause death after 1-year follow-up, according to vascular disease.

| **ENDPOINT** | | **PRIMARY EFFECT ESTIMATES** | | | |
| --- | --- | --- | --- | --- | --- |
| **Ischemic stroke** | | **Crude HR**  **(95% CI)** | | **Adjusted HR***  **(95% CI)** | |
|  | |  |  |  |  |
|  | PAD vs. no vascular disease | 1.78 | (1.44 to 2.21) | 1.29 | (1.04 to 1.61) |
|  | Prior MI vs. no vascular disease | 1.12 | (0.96 to 1.29) | 1.02 | (0.88 to 1.18) |
|  | PAD vs. prior MI | 1.60 | (1.26 to 2.03) | 1.30 | (1.02 to 1.66) |
|  |  |  |  |  |  |
| **All-cause death** | | **Crude HR**  **(95% CI)** | | **Adjusted HR***  **(95% CI)** | |
|  | |  |  |  |  |
|  | PAD vs. no vascular disease | 1.51 | (1.39 to 1.64) | 1.46 | (1.34 to 1.59) |
|  | Prior MI vs. no vascular disease | 0.85 | (0.80 to 0.90) | 0.94 | (0.86 to 1.00) |
|  | PAD vs. prior MI | 1.77 | (1.61 to 1.95) | 1.53 | (1.39 to 1.68) |
|  |  |  |  |  |  |
| (Abbreviations: HF: heart failure; HR: hazard rate ratio; MI: myocardial infarction; PAD: peripheral artery disease; 95% CI: 95% confidence interval)  *Adjusted for sex (binary), hypertension (binary), diabetes (binary), prior stroke/transient ischemic attack (binary), COPD (binary), renal disease (binary), and age (continuous) | | | | | |
