# Supplementary material for: Vascular Disease and Risk Stratification for Ischemic Stroke and All-Cause Death in Heart Failure Patients without Diagnosed Atrial Fibrillation: A Nationwide Cohort Study
Source: PLoS One. 2016 Mar 25;11(3):e0152269. doi: 10.1371/journal.pone.0152269 (PMC4807813; doi:10.1371/journal.pone.0152269)
Supplement: S6 Table — (DOCX) [file pone.0152269.s007.docx]

**S6 Table.** Sensitivity analysis excluding patients with a history of stroke: Hazard rate ratios of incident stroke after 1-year follow-up, according to vascular disease.

| **ENDPOINT** | | **PRIMARY EFFECT ESTIMATES** | | | | |
| --- | --- | --- | --- | --- | --- | --- |
| **Ischemic stroke** | | **Crude HR**  **(95% CI)** | | **Adjusted HR***  **(95% CI)** | | |
|  | |  |  |  |  | |
|  | PAD vs. no vascular disease | 1.68 | (1.42 to 2.00) | 1.52 | (1.28 to 1.81) | |
|  | Prior MI vs. no vascular disease | 1.09 | (0.99 to 1.21) | 1.09 | (0.98 to 1.21) | |
|  | PAD vs. prior MI | 1.54 | (1.28 to 1.86) | 1.38 | (1.14 to 1.67) | |
|  |  |  |  |  |  | |
| (Abbreviations: HF: heart failure; HR: hazard rate ratio; MI: myocardial infarction; PAD: peripheral artery disease; 95% CI: 95% confidence interval)  *Adjusted for sex (binary), hypertension (binary), diabetes (binary), prior stroke/transient ischemic attack (binary), COPD (binary), renal disease (binary), and age (continuous) | | | | | |  |
