# Supplementary material for: Vascular Disease and Risk Stratification for Ischemic Stroke and All-Cause Death in Heart Failure Patients without Diagnosed Atrial Fibrillation: A Nationwide Cohort Study
Source: PLoS One. 2016 Mar 25;11(3):e0152269. doi: 10.1371/journal.pone.0152269 (PMC4807813; doi:10.1371/journal.pone.0152269)
Supplement: S7 Table — (DOCX) [file pone.0152269.s008.docx]

**S7 Table.** Relative risks of ischemic stroke and all-cause death after 1-year follow-up, according to vascular disease.

| **ENDPOINT** | | **PRIMARY EFFECT ESTIMATES** | | | |  |
| --- | --- | --- | --- | --- | --- | --- |
| **Ischemic stroke** | | **Crude RR**  **(95% CI)** | | **Adjusted RR***  **(95% CI)** | |  |
|  | |  |  |  |  |  |
|  | PAD vs. no vascular disease | 1.69 | (1.38 to 2.08) | 1.10 | (0.84 to 1.45) |  |
|  | Prior MI vs. no vascular disease | 1.11 | (0.96 to 1.28) | 0.95 | (0.78 to 1.15) |  |
|  | PAD vs. prior MI | 1.53 | (1.22 to 1.93) | 1.18 | (0.82 to 1.69) |  |
|  |  |  |  |  |  |  |
| **All-cause death** | | **Crude RR**  **(95% CI)** | | **Adjusted RR***  **(95% CI)** | |  |
|  | |  |  |  |  |  |
|  | PAD vs. no vascular disease | 1.43 | (1.34 to 1.54) | 1.32 | (1.23 to 1.41) |  |
|  | Prior MI vs. no vascular disease | 0.86 | (0.82 to 0.91) | 0.99 | (0.94 to 1.04) |  |
|  | PAD vs. prior MI | 1.67 | (1.54 to 1.80) | 1.20 | (1.10 to 1.30) |  |
|  |  |  |  |  |  |  |
| (Abbreviations: HF: heart failure; MI: myocardial infarction; PAD: peripheral artery disease; RR: relative risk; 95% CI: 95% confidence interval)  *Adjusted for sex (binary), hypertension (binary), diabetes (binary), prior stroke/transient ischemic attack (binary), COPD (binary), renal disease (binary), and age (continuous) | | | | | | |
