# Supplementary material for: Vascular Disease and Risk Stratification for Ischemic Stroke and All-Cause Death in Heart Failure Patients without Diagnosed Atrial Fibrillation: A Nationwide Cohort Study
Source: PLoS One. 2016 Mar 25;11(3):e0152269. doi: 10.1371/journal.pone.0152269 (PMC4807813; doi:10.1371/journal.pone.0152269)
Supplement: S8 Table — (DOCX) [file pone.0152269.s009.docx]

**S8 Table.** Relative risks of ischemic stroke and all-cause death after 5-years follow-up, according to vascular disease.

| **ENDPOINT** | | **PRIMARY EFFECT ESTIMATES** | | | |  |
| --- | --- | --- | --- | --- | --- | --- |
| **Ischemic stroke** | | **Crude RR**  **(95% CI)** | | **Adjusted RR***  **(95% CI)** | |  |
|  | |  |  |  |  |  |
|  | PAD vs. no vascular disease | 1.35 | (1.16 to 1.56) | 0.98 | (0.82 to 1.17) |  |
|  | Prior MI vs. no vascular disease | 1.16 | (1.06 to 1.27) | 1.09 | (0.98 to 1.21) |  |
|  | PAD vs. prior MI | 1.16 | (0.99 to 1.36) | 0.85 | (0.70 to 1.05) |  |
|  |  |  |  |  |  |  |
| **All-cause death** | | **Crude RR**  **(95% CI)** | | **Adjusted RR***  **(95% CI)** | |  |
|  | |  |  |  |  |  |
|  | PAD vs. no vascular disease | 1.36 | (1.31 to 1.42) | 1.24 | (1.20 to 1.29) |  |
|  | Prior MI vs. no vascular disease | 0.88 | (0.86 to 0.91) | 0.95 | (0.93 to 0.98) |  |
|  | PAD vs. prior MI | 1.54 | (1.47 to 1.61) | 1.27 | (1.22 to 1.32) |  |
|  |  |  |  |  |  |  |
| (Abbreviations: HF: heart failure; MI: myocardial infarction; PAD: peripheral artery disease; RR: relative risk; 95% CI: 95% confidence interval)  *Adjusted for sex (binary), hypertension (binary), diabetes (binary), prior stroke/transient ischemic attack (binary), COPD (binary), renal disease (binary), and age (continuous) | | | | | | |
