# Supplementary material for: Vascular Disease and Risk Stratification for Ischemic Stroke and All-Cause Death in Heart Failure Patients without Diagnosed Atrial Fibrillation: A Nationwide Cohort Study
Source: PLoS One. 2016 Mar 25;11(3):e0152269. doi: 10.1371/journal.pone.0152269 (PMC4807813; doi:10.1371/journal.pone.0152269)
Supplement: S1 Text — (DOCX) [file pone.0152269.s010.docx]

**S1 Text. Methodological details.** Analysis on a risk-scale.

In order to have the complete risk assessment of vascular disease in HF patients, both rates and relative risks are needed. Thus, regression analysis was used to compare the 1- and 5-years relative risks of the endpoints according to the presence of PAD or prior MI. To this end, we used generalized linear regression alongside the pseudovalue method in order to take into account the competing risks of death (Klein J, Andersen P. Regression modeling of competing risks data based on pseudovalues of the cumulative incidence function. *Biometrics*. 2005;61:223–229; Klein J, Logan B, Harhoff M, Andersen P. Analyzing survival curves at a fixed point in time. *Stat Med*. 2007;26:4505–4519). The pseudo-value regression technique reduces to simple regression (with a log-link function) on the event status indicator at 1 year in the absence of censoring, whereas censored observations (for which the event status is not observed) are replaced with pseudo-observations based upon Aalen-Johansen cumulative incidence estimates using the jackknife method.
